# Evaluation of the effect of the indoor environment on the physiological responses of early-gestation sows in a commercial house in China

**DOI:** 10.3389/fvets.2023.1178970

**Published:** 2023-06-01

**Authors:** Yangyang Li, Tong Li, Bin Shang, Yang Zhao, Xiuping Tao, Feng Peng, Xiaojun Zou, Sixin Zhang

**Affiliations:** ^1^Key Laboratory of Energy Conservation and Waste Management in Agricultural Structures, Institute of Environment and Sustainable Development in Agriculture, Chinese Academy of Agricultural Sciences, Beijing, China; ^2^Institute of Urban Agriculture, Chinese Academy of Agricultural Sciences, Chengdu, China; ^3^Department of Animal Science, The University of Tennessee, Knoxville, Knoxville, TN, United States; ^4^Henan Zhumei Swine Breeding Co., Ltd., Zhumadian, China; ^5^Zhengyang Pig Farm in Henan Province, Zhumadian, China

**Keywords:** physiology, sow, temperature humidity index, threshold, index

## Abstract

**Objective:**

The environment influences the sow's health and physiology during gestation. This study was conducted to evaluate indoor environmental parameters and physiological responses of early-gestation sows and investigate the possible methods for assessing the thermal environment in commercial houses.

**Methods:**

A total of 20 early-gestation sows (commercial purebred Yorkshire) with an average body weight of 193.20 ± 3.62 kg were used for this study in winter, spring, summer, and autumn. The indoor environment parameters comprising dry-bulb temperature (T_db_), relative humidity (RH), and carbon dioxide (CO_2_) were recorded in 30-min intervals. Physiological parameters including heart rate (HR) and respiration rate (RR) of sows were also measured every 30 min. Wet-bulb temperature (T_wb_) was calculated using T_db_, RH and atmospheric pressure was recorded at a nearby weather station.

**Results:**

The average indoor T_db_ and RH were 12.98 ± 2.03°C and 80.4 ± 6.4% in winter, 18.98 ± 2.68°C and 74.4 ± 9.0% in spring, 27.49 ± 2.05°C and 90.6 ± 6.4% in summer, and 17.10 ± 2.72°C and 64.5 ± 10.9% in autumn. A higher average concentration of CO_2_ was observed in winter (1,493 ± 578 mg/m^3^) than in spring (1,299 ± 489 mg/m^3^), autumn (1,269 ± 229 mg/m^3^), and summer (702 ± 128 mg/m^3^). Compared with the HR and RR in the optimum environment, high RH in the house led to a significant decrease in both HR and RR (*P* < 0.05). In addition, a significant decline in HR was also obtained at high temperatures (*P* < 0.05). A temperature humidity index (THI), THI = 0.82 × T_db_ + 0.18 × T_wb_, was determined for early-gestation sows, and the THI thresholds were 25.6 for HR. The variation in THI in summer showed that heat stress still occurred under the pad-fan cooling system.

**Conclusion:**

This study demonstrated the critical significance of considering physiological responses of early-gestation sows in commercial houses and THI thresholds. We recommend that much more cooling measures should be taken for early-gestation sows in summer.

## 1. Introduction

It is well-known that China is the largest pig producer and pork consumer in the world. Even under the influence of African Swine Fever in 2019, annual pork production reached 42.55 million tons, with the average profit value of a commercial pig being 21 times more than that of 2018 (1). However, the prolonged and adverse effects of indoor air quality and thermal environment are well known to have significant implications for intensive pig production in commercial houses (2–4), resulting in impaired health and welfare and even death (5–8). Particularly, heat stress (HS) caused by elevated temperature (32°C) has been widely recognized as one of the greatest challenges in the pig industry, which affects feed efficiency, weight gain, reproduction, and welfare (9–11). As a result, it is imperative to alleviate HS and maintain optimal environment surroundings for the animals for sustainable and intensive pig production (12).

Gestation sows are critical to confined animal feeding operations since the quality and development of fetal growth were dependent on the health and strength of sows. The sows are sensitive to HS due to the limited number of sweat glands and the presence of back fat. However, sows are sensitive to HS (5, 6, 13), which could lead to decreased feed intake, anestrus, and farrowing rates, increased embryonic mortality of late-gestation sows, and declined growth and viability of piglets (6, 12, 14–17). The impaired reproduction performance of late-gestation sows might be attributed to the physiological responses under acute and cyclic high-temperature (28–32°C) conditions (5, 18). Only a few studies regarding the impacts of thermal environments on early-gestation sows have been published, which mainly focused on behavior, gut microbiota, placental efficiency, and fetal development (19). In addition, as an important indicator for assessing indoor air quality, carbon dioxide (CO_2_) is another gas contaminant produced in confined pig houses, which is also a useful tool to determine the ventilation rate for pig production (20, 21). CO_2_ also limits weight gain, behavior, vocalizations, and muscular excitation (22). Within this context, the impact of the indoor environment represents a critical issue for consideration in ensuring the health and welfare of pigs in confined buildings.

In pig production, several cooling methods have been widely used to improve animal comfort during hot weather. In particular, an increasing body of studies has implicated that the heat was removed effectively via different cooling technologies combining water evaporation and high forced air velocity, together with floor cooling (23). Previous studies also claimed that the cooling measures led to a reduction in respiratory rate and skin temperature and a higher level of feed intake (23–25). Taking into consideration the intensive pig production systems in China, the swine houses are typically made of brick with sidewall windows; the windows are open for ventilation under mild climates, which could reduce the energy consumption of ventilation, and pad-fan cooling systems are preferred in the summer. In recent years, researchers have investigated the cooling efficiency and environmental characteristics of swine barns with pad-fan cooling systems. Meanwhile, the effect of pad-fan cooling systems on sows' physiological responses was also explored under hot conditions (26, 27). However, there is a lack of research concerning HS alleviation of sows under hot conditions in commercial pig productions. As a cooling method, the effects of pad-fan systems on sows' HS need further investigation. We hypothesized that the utilization of pad-fan systems in commercial houses in China could not be effective in reducing the HS of sows. Therefore, in our study, we evaluated the cooling effect of pad-fan cooling systems in a swine house in the summer, delineated the physiological responses of early-gestation sows under commercial conditions in different seasons, and explored the HS assessment method for sows under commercial pig production conditions.

## 2. Materials and methods

### 2.1. Animals and housing

All of the animal handling involving sows was approved by the Animal Welfare Committee of the Institute of Environment and Sustainable Development in Agriculture, Chinese Academy of Agriculture Sciences (IEDACAAS, 20201201).

**Figure 1:**
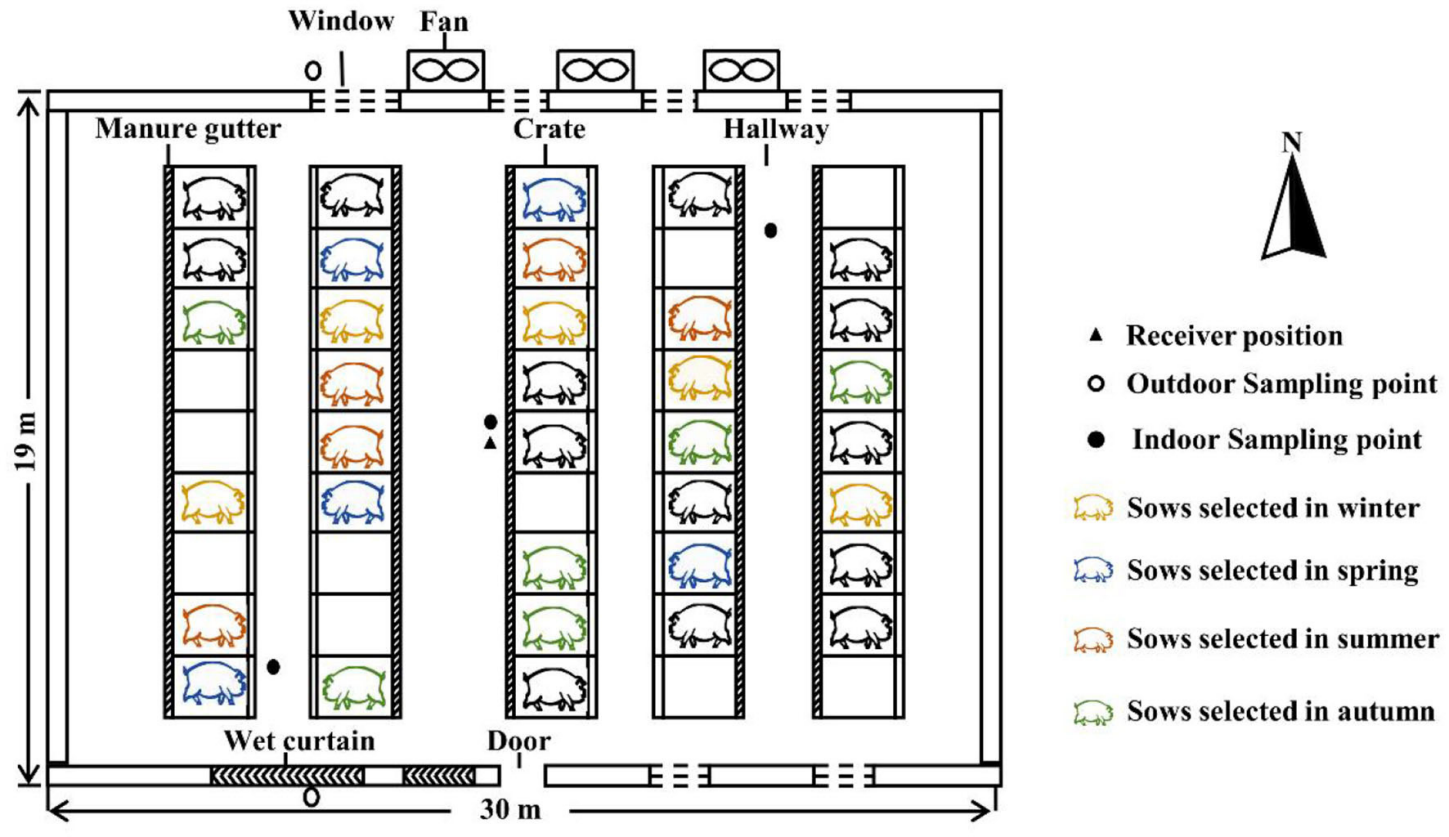
Schematic drawing of the sow barn (top view) and sampling locations. Twenty sows (out of 35) were selected for testing in winter, spring, summer, and autumn (five sows in each season). Signals of physiological parameters were recorded by a receiver positioned in the middle of the hallway.

This study was conducted on a pig-breeding farm (Henan, China). There were five parallel pig pens on the north and south sides of the building. Each pen was comprised with nine individual crates, with fully slatted concrete floors, which had a capacity of housing 45 sows (Figure 1). Separate feeders and drinkers were equipped in each crate. Feces, urine, and wastewater were stored in a manure gutter (16.2 m × 0.3 m × 0.3 m, length × width × deepth) beneath the pen and were removed once a week. Air flowed into the barn through ventilation windows in spring and autumn, and the windows were closed in winter and summer. A mechanical ventilation system with three exhaust fans and a cooling pad system (wet curtain) was used in the summer. The fan had two operating options (off or 100% speed), and it was running at 100% speed during summer treatment.

The experiments were performed in winter (21 January to 26 February), spring (6 April to 13 May), summer (5 July to 3 August), and autumn (12 October to 15 November), 2021. Taking into consideration the commercial conditions in the commercial swine barn, a total of five multiparous and healthy sows (commercial purebred Yorkshire) were randomly selected in each season. The sows were artificially inseminated and confirmed pregnant, and then, the trials began and lasted for 30–35 days. The five sows were draped in a vest tailored to their size. Before the experiment, the selected sows were allowed to acclimatize to the tailored vest for 5 days after mating. Throughout the experiment, all the sows in the barn were limit-fed (2 kg/day) a diet, to meet or exceed nutrient requirements. Meanwhile, sows had *ad libitum* access to water, and the daily diet was divided into two meals at ~09:00 and 15:00. The body weight and parity of sows in the experiment are shown in Supplementary Table S1.

### 2.2. Environmental condition

There were three indoor sampling locations in this study. Indoor dry-bulb temperature (T_in_) and relative humidity (RH_in_) were recorded with three logger devices (Hobo; accuracy ±0.10°C and 3% for T_db_ and relative humidity, respectively; Onset UX100-003; Bourne, MA, USA). These three data loggers were situated at a height of 0.8 m above the floor at sows standing level, away from water sources. Meanwhile, two different data loggers (TS-WD; data logger temperature/RH; accuracy ±0.21°C and 3.5% for temperature and relative humidity, respectively; Zhengzhou TOLES Technology Co., Ltd, Henan, China) were installed at a level of 1.8 m outside the north and south windows to avoid the influence of sunlight on the outdoor dry-bulb temperature (T_out_) and relative humidity (RH_out_).

The indoor CO_2_ concentration was monitored using a gas detector (accuracy ±2% F.S, respectively; MIC-600, Shenzhen Eranntex electronics CO., LTD., Shenzhen, China), and the data were recorded at an interval of 30 min. The calibration procedure was performed every 2 weeks with standard gases (2,650 ppm for CO_2_; National Institute of Metrology, Beijing, China).

### 2.3. Physiological measurements

After a 5-day acclimation, the physiological responses (heart rate and respiration rate) of sows were recorded at an interval of 30 min using the Jacketed External Telemetry system (accuracy ±0.01V, Data Sciences International, St. Paul, MN, USA). The sampling sites are presented in Supplementary Figure S1.

To avoid the variation of physiological responses caused by feed intake, the differences in physiological parameters were compared at 0 h (feeding), 0.5 h (0.5 h after feeding), 1 h (1 h after feeding), 1.5 h (1.5 h after feeding), and 2 h (2 h after feeding). The variations in physiological responses after feeding are shown in Supplementary Table S2. According to the results of the comparison, the data of 0 h (feeding), 0.5 h (0.5 h after feeding), and 1 h (1 h after feeding) were excluded when evaluating the influence of the environment on sows.

### 2.4. Statistics

All the statistical analyses were performed using SPSS (version 21, IBM, Armonk, NY, USA). In this study, the data were first tested for the normal distribution with the Shapiro–Wilk test and the log10 transformation occurred for those that did not pass the test. Based on the season, the environmental parameters and physiological responses of sows were analyzed using repeated analysis of variance (ANOVA) to determine the differences among the subjects at all observation times (30 min). Statistical comparisons were made using Tukey–Kramer testing, and the significance was set at P ≤ 0.05. Tukey–Kramer multiple comparison tests were performed to compare the mean values, and all of the environmental and physiological parameters were represented as mean ± standard deviation (SD).

#### 2.4.1. Correlation between environmental factors and physiological responses

A canonical correlation analysis (CCA) was applied to quantify the relationships between environmental parameters and physiological responses, and the detailed calculation of CCA was described by Xie et al. (28).

#### 2.4.2. Quantitative relationship between the thermal environment and physiological indices

The thermoneutral zone (TNZ) of gestation sows was 15–20°C and 60%−70% of relative humidity (29). The HR means of sows under the TNZ were averaged as the baseline. The theoretical thermodynamic wet-bulb temperature (T_wb_) was calculated using recommended equations described previously (30).

The HR and RR of sows at T_in_ more than 20°C were sorted out and compared with the corresponding baseline to obtain the variation of physiological indices, i.e., ΔHR and ΔRR. The weighting factors of dry-bulb temperature (T_db_) varied from 0 to 1, and correlation coefficients between assumed THI and physiological parameters (HR and RR) were calculated. A method of multiple regression analyses was conducted to determine the temperature–humidity index, incorporating temperature and humidity, which could describe the quantitative relationships between the thermal environment and physiological responses. Hence, the weighting factor (a) of T_db_ was determined when the quadratic regression model was solved.

The relationship between THI and the physiological parameters was performed to identify the THI thresholds at which HR and RR begin to increase or decrease. The THI thresholds were determined using a 2-phase segmented regression with the piecewise procedure using OriginPro 2022b (OriginLab Corp., Northampton, MA, United States).

## 3. Results

### 3.1. Thermal and gaseous environment condition

**Figure 2:**
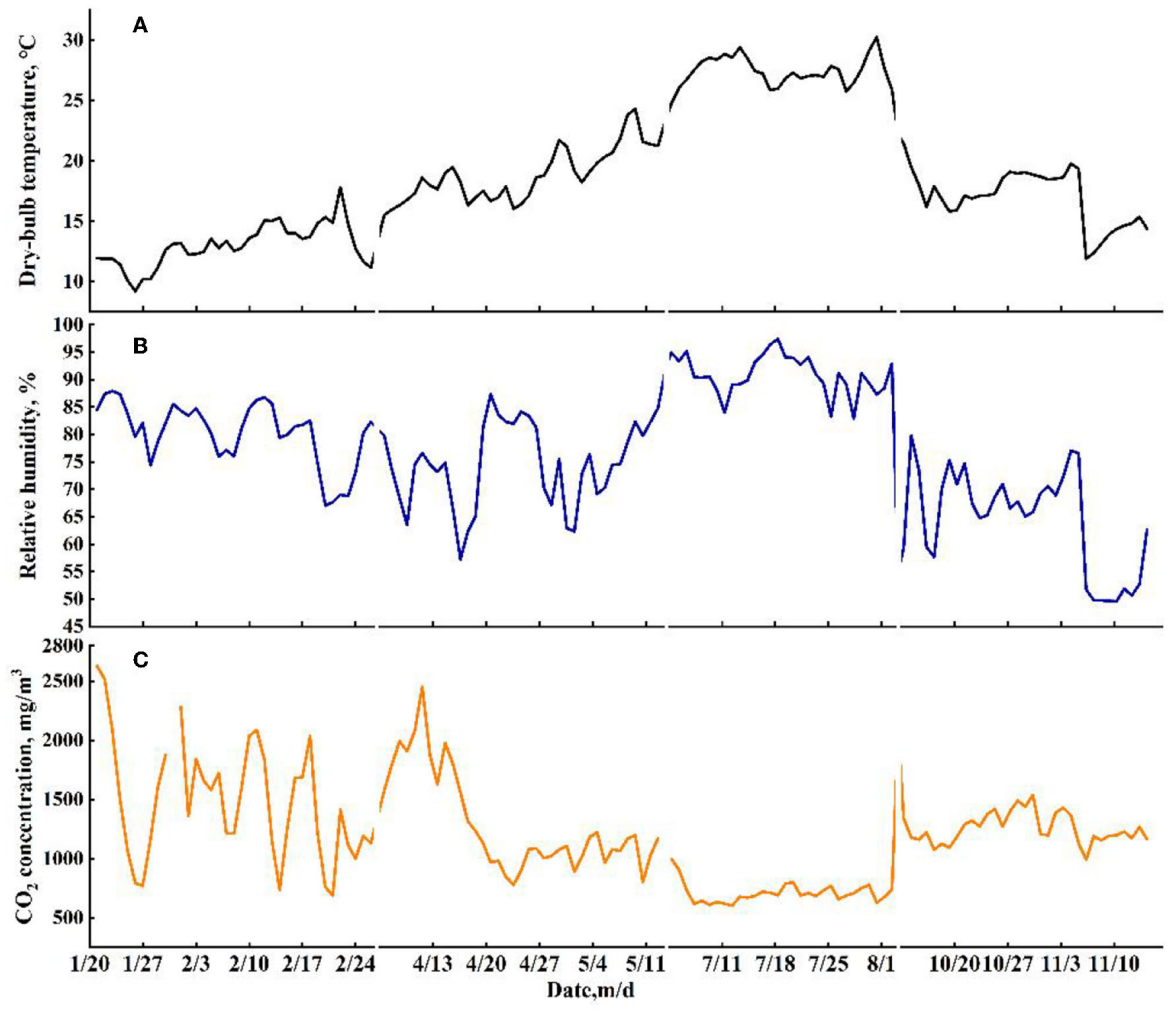
The profiles of daily averaged dry-bulb temperature **(A)**, relative humidity **(B)**, and CO_2_ concentration **(C)** during the experimental period.

The profiles of daily averaged T_in_ and RH_in_ during experimental periods are shown in Figures 2A, B. A larger variation in indoor T_in_ was observed in autumn (from 11.90 to 22.17°C) than that in winter (from 9.19 to 17.81°C), spring (from 15.52 to 24.29°C), and summer (from 23.55 to 30.23°C). The indoor RH_in_ had a larger range and fluctuation in spring (from 57.2 to 87.3%) and autumn (from 49.6 to 79.8%) than the RH_in_ in winter (from 67.0 to 87.9%) and summer (from 82.9 to 97.4%). The variations in CO_2_ concentration are shown in Figure 2C. Compared with the indoor CO_2_ concentration in winter, spring, and autumn, the smallest fluctuations were found in summer, which ranged from 603 to 998 mg/m^3^.

**Figure 3:**
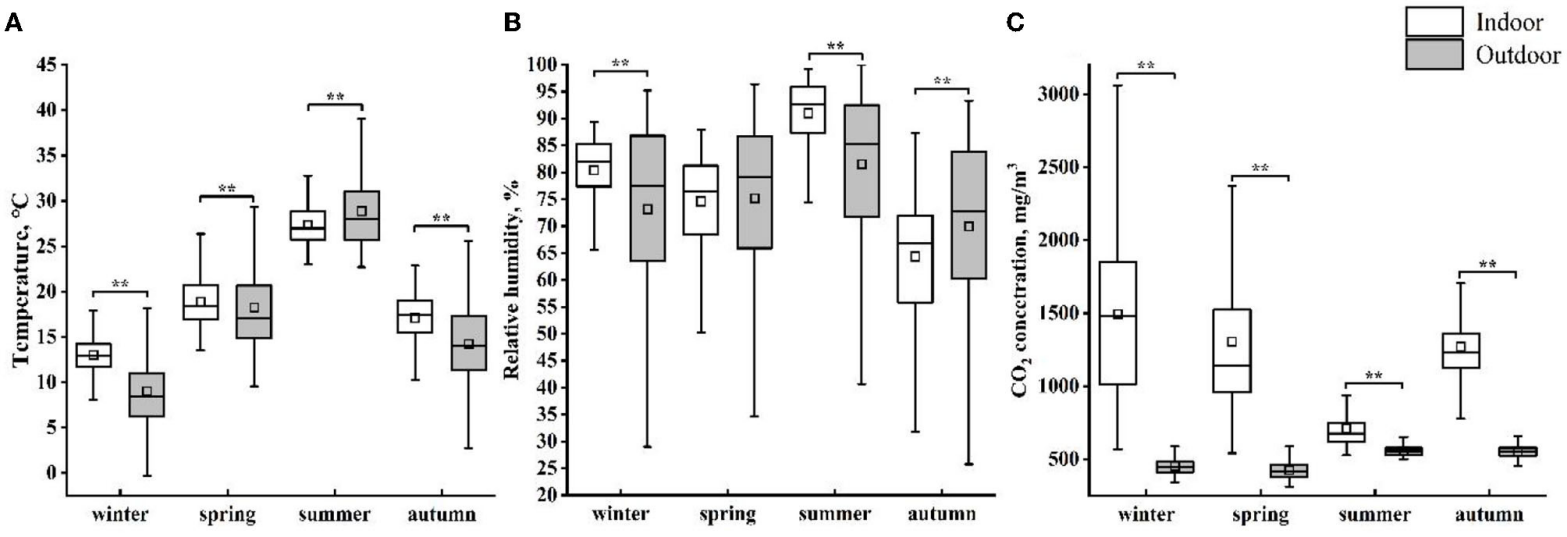
The indoor and outdoor averages of temperature **(A)**, relative humidity **(B)**, and CO_2_ concentration **(C)** for each season. The plot shows mean (square box), median (line within box), 25th and 75th percentiles (box), and 5th and 95th percentiles (whiskers). ** indicates a significant difference (*P* < 0.01) between indoor and outdoor environmental parameters in each season.

The seasonal average environmental parameters (indoor and outdoor) are shown in Figure 3. There is a significant difference in temperature both inside and outside the house, and the seasonal average T_in_ was significantly lower than T_out_ (*P* < 0.01). In spring, no difference was found between RH_in_ and RH_out_. In each season, the CO_2_ concentration inside the barn was higher than the outdoor CO_2_ concentration (*P* < 0.01).

### 3.2. Physiological responses under commercial environmental conditions

The HR means were 85.44 ± 9.54 beats per min (bpm), 80.88 ± 8.34 bpm, 71.01 ± 9.74 bpm, and 86.02 ± 12.85 bpm in winter, spring, summer, and autumn, respectively. The peak HR of 123.06 bpm appeared in autumn, and the valley value of 50.00 bpm was observed in summer. The sows' RR means were 13.80 ± 6.79 breaths per min (brpm), 13.03 ± 5.66 brpm, 16.39 ± 10.48 brpm, and 14.81 ± 8.83 brpm in spring, summer, and autumn, respectively. The averaged values of HR and RR within and beyond the TNZ are shown in Table 1. The HR of sows under the TNZ was significantly higher than that under the environment beyond the TNZ (*P* < 0.001). A significant difference (*P* < 0.05) in RR was also observed between humidity within and beyond the TNZ.

**Table 1:** Heart rate and respiration rate of early-gestation sows exposed to a different environment.

**Item**	**Dry-bulb temperature**			**Humidity**		
	**Within TNZ**	**Beyond TNZ**	***P*** **-value**	**Within TNZ**	**Beyond TNZ**	***P*** **-value**
HR	81.78 ± 8.15	75.73 ± 8.69	<0.001	82.36 ± 8.65	77.58 ± 8.69	<0.001
RR	13.15 ± 4.81	13.27 ± 5.18	>0.05	13.93 ± 5.45	12.85 ± 4.76	<0.05

TNZ, Thermoneutral zone, the recommended temperature of 15–20°C and relative humidity of 60–70%; HR, heart rate (beat per min, bpm); RR, respiration rate (breath per min, brpm).

Significant at P ≤ 0.05.

### 3.3. Correlation of environmental factors with physiological responses

The result of CCA between independent variables (indoor environmental parameters) and dependent variables (physiological responses) is shown in Table 2, and two pairs of canonical variables were identified (*P* < 0.001). According to the percentage of variation in two pairs of canonical variables, the first pair of canonical variables could represent well the relationship between independent and dependent variables. Canonical loadings and cross-loadings of the first pair of canonical variables indicated that the T_in_ and RH_in_ were strongly correlated with their first canonical variate (Table 3). The first pair of canonical variates was also characterized by a strong canonical loading on HR. T_in_, RH_in_, and HR had the highest correlations in the first canonical variables, which were considered the major reference for the evaluation of the indoor environment.

**Table 2:** Canonical correlations and overall model fit.

**Canonical function**	**Canonical correlation**	**Eigenvalues**	**Percentage of variation**	**Probability**
1	0.496	0.326	0.851	<0.001
2	0.232	0.057	0.149	<0.001

**Table 3:** Standardized canonical coefficients, loadings, and cross-loading.

**Canonical variables**		**Standardized canonical coefficients**		**Canonical loadings**		**Cross loadings**	
		**1**	**2**	**1**	**2**	**1**	**2**
Independent	T_in_	−0.880	0.814	0.966	0.203	0.479	0.047
	RH_in_	0.141	−1.160	0.743	0.579	0.368	−0.135
	CO_2_	0.039	−0.172	−0.627	−0.038	0.311	−0.009
Dependent	HR	−1.012	−0.021	−0.991	0.133	−0.491	0.031
	RR	0.135	1.003	−0.021	1.000	−0.010	0.232

T_in_, indoor dry-bulb temperature; RH_in_, indoor relative humidity; CO_2_, indoor CO_2_ concentration; HR, heart rate; RR, respiration rate.

### 3.4. The relationship between thermal environment and physiological indices

The THI based on HR for early-gestation sows was fitted as the following equation:


(1) $$\begin{array}{l} \text{THI}=0.82\times {\text{T}}_{\text{db}}+0.18\times {\text{T}}_{\text{wb}} \end{array}$$

**Figure 4:**
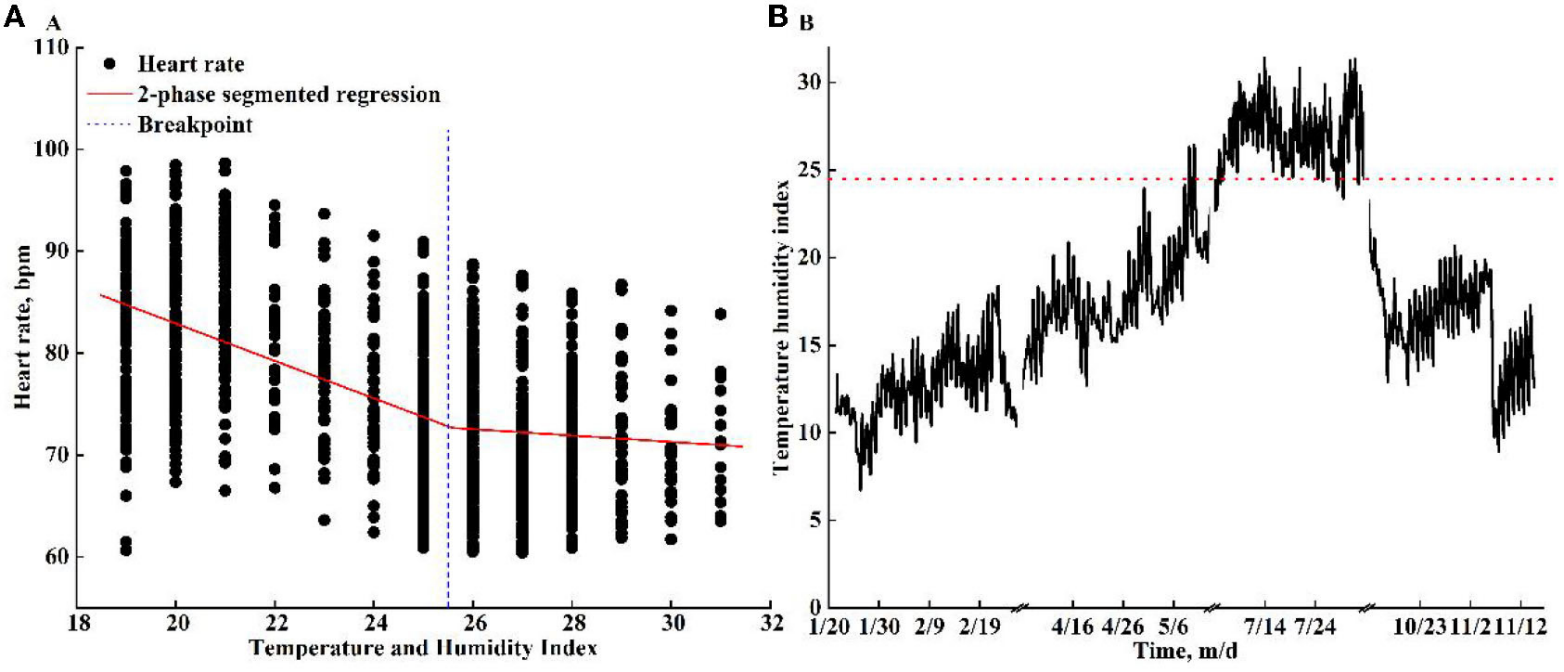
The variation of heart rate (beats per min, bpm) when the temperature–humidity index (THI) ranged from 18 to 32 **(A)** and THI **(B)** in each season. A breakpoint (blue vertical dot line) was detected at 25.6 THI for heart rate. The red horizontal dotted line indicates the THI threshold.

The relationships between THI and HR were plotted (Figure 4A). The THI varied from 18 to 32 during the treatment. The results of the two-phase segment regression indicated a breakpoint (THI threshold) at 25.6 THI (95% CI: 24.7–26.5, Figure 4A). There was an overall decline in HR as the THI increased, and the HR dropped slightly when THI was >25.6. The THI for each season is presented in Figure 4B, the THI threshold indicated that HS occurred in the summer under the pad-fan cooling system. The time proportion of the THI raised above the threshold in summer was 70.95%.

## 4. Discussion

The indoor T_in_, RH_in_, and CO_2_ concentrations varied greatly in four seasons, which were affected by the management and structure of buildings, ventilation control, stock density, season, and outside weather conditions (3). The larger fluctuations of T_in_, RH_in_, and CO_2_ concentration in spring and autumn than that in winter and summer could be attributed to the varied weather outside the house (31, 32). The smallest variations in T_in_ and RH_in_ were observed in summer in comparison with those values in spring and autumn due to the pan-fan cooling systems in summer. Several studies indicated that variations in ambient temperature, as well as the activity, weight of pigs, and ventilation control, would all have a significant impact on CO_2_ concentrations in pig houses (33–35). The relatively low CO_2_ readings in summer might be attributed to the mechanical ventilation coupled with the wet-pad system.

The HR of gestation sows under the TNZ was found in this study to be slightly higher than the values of 70–80 bpm (36), which might be attributed to the larger weight and litter sizes of modern pigs (37). A declining trend was observed in HR with increasing T_in_ and RH_in_. Our result did not agree with the study by Parois et al. (38), who reported an increased HR tendency of late lactation sows exposed to acute HS. Some studies proved that the HR of pigs was raised to support the increase in blood flow to the skin, which was beneficial to maintaining euthermia in hot environments (39). On the contrary, another research reported a decrease in HR when pigs were exposed to hot environments (40). Previous literature proved that HR and its rhythm were mainly regulated by the sympathetic nervous system and the parasympathetic nervous system (41). The method of heart rate variability (HRV), including time domain analysis and frequency domain analysis, was developed to explore the effect of HS on HR. The results of HRV indicate that the HRV decreased due to the increase in sympathetic excitability in hot environments, which accounted for the decline in HR (42). This may explain the overall decrease in HR when gestation sows are exposed to the hot environment in the present study.

As a result of increased fetal growth and development, gestation sows would produce an increase in metabolic heat production (13), which may lead to an increased heat load (43). Hence, greater heat loss efforts through skin vasodilation and increased RR would be required for sows under gestation to maintain euthermia. The average RR of gestation sows increased by 10 brpm per °C when the ambient temperature was higher than 20°C (15). In this present study, higher RR was observed in sows under hot temperatures, which was consistent with previous studies (16, 44, 45). Animals have evolved coping strategies to mitigate the effects of external stresses when exposed to a continued stressor (46), and the increase in RR is likely to be related to the duration of heat treatment (19). Liu et al. (47) reported an average RR of 21.54 brpm for gestation sows exposed to chronic HS, which was a bit higher than that in our research.

Temperature and humidity play essential roles in total heat exchange between animals and their environments. The combination of T_in_ and RH_in_ is effective at assessing the thermal environment (48), and the THI was proven to be an effective index that reflects the animal's response to the thermal environment (49). The THI was expressed by the equation THI = 0.65 × T_db_ + 0.35 × T_wb_ for young pigs (10–15 kg) and THI = 0.75 × T_db_ + 0.25 × T_wb_ for fattening or finishing pigs weighing from 70 to 120 kg (50, 51). In our study, the weight of 0.82 for T_db_ was obtained for early-gestation sows. A comparison of the findings with other studies confirmed that the thresholds were determined, and body temperature increased rapidly when THI>28 for pigs at a weight of 12 kg (52). Some other THIs combining temperature and relative humidity were also proposed to assess the impact of the thermal environment on pigs. Lucas et al. (53) reported that HS occurred when the index value reached 75. In addition, based on the coefficient of determination for different heat load functions, the THI thresholds in the crossbreed data were 67 in Missouri, 72 in North Carolina, and 70 in North Carolina for pure breeds (54). Regarding the relationship between pregnancy rates, the results indicated that the THI threshold for sows was 78 (55). However, unlike studies related to cattle, there are few studies to determine THI thresholds in sows based on the correlation between THI and physiological responses. The finding of the THI thresholds determined in the current study was lower than in previous research. A possible explanation for a lower threshold in our research is that the sows selected in our experiment had a greater live weight (193.20 ± 3.62 kg) than pigs in previous research. It has been reported that pigs with higher live weight were more sensitive to HS, which compromised their feed intake, growth performance, and reproductive performance (56, 57). Moreover, sows under gestation were more vulnerable to HS (6, 58–60). In addition, the percentage above the THI threshold was as high as 70.95, implying that the pad-fan cooling system could not tackle the HS of sows incurred in summer weather, the cooling efficiency of the pad-fan cooling system should be improved, or other efficient measures should be sought in summer.

## 5. Conclusion

In this study, we evaluated the indoor thermal and gas environment in a commercial pig house for four seasons and assessed the physiological responses of early-gestation sows. There were large variations in temperature, relative humidity, and CO_2_ concentration in both spring and autumn. Higher RR and lower HR of early-gestation sows were exhibited in summer. Furthermore, the THI thresholds of early-gestation sows were 25.6 for HR. These THI thresholds imply that the HS of early-gestation sows occurred under pad-fan cooling systems, and much more efficient cooling measures should be taken in commercial houses. These thresholds can be served as a basis to develop HS mitigation techniques for early-gestation sows.

## Data availability statement

The original contributions presented in the study are included in the article/Supplementary material, further inquiries can be directed to the corresponding author.

## Ethics statement

The animal study was reviewed and approved by Ethics Committee, Institute of Urban Agriculture, Chinese Academy of Agricultural Sciences.

## Author contributions

YL: conceptualization, methodology, writing—original draft, and visualization. TL: methodology, validation, and resources. BS: methodology, software, and validation. YZ: writing—reviewing and editing. XT: methodology, validation, writing—reviewing and editing, and supervision. FP and SZ: resources. XZ: resources and investigation. All authors contributed to the article and approved the submitted version.
