# Supplementary material for: Evaluation of the effect of the indoor environment on the physiological responses of early-gestation sows in a commercial house in China
Source: Front Vet Sci. 2023 Jun 1;10:1178970. doi: 10.3389/fvets.2023.1178970 (PMC10270292; doi:10.3389/fvets.2023.1178970)
Supplement: Supplementary file 1 [file Data_Sheet_1.pdf]

## Supplementary Files

## Supplemental Table S1

The body weight and parity of sows in each season in this experiment (Mean±SD)

| | Season | | | | *P*-valus |
|---|---|---|---|---|---|
| | **winter** | **spring** | **summer** | **autumn** | |
| Body Weight[a], Kg | 190.16±7.41 | 191.13±4.49 | 192.17±3.81 | 199.34±5.79 | 0.12 |
| parity | 3.24±0.37 | 3.18±0.53 | 3.22±0.34 | 3.34±5.79 | 0.96 |

## Supplemental Table S2

The variation of thermal environment and physiological responses of gestation sows after feeding

| **Parameter** | **0h** | **0.5h** | **1h** | **1.5h** | **2h** |
|---|---|---|---|---|---|
| $T_{db}$ | 23.56±5.02 | 23.72±4.98 | 23.77±4.91 | 23.88±4.87 | 23.96±4.85 |
| RH | 79.61±11.85 | 79.54±11.69 | 79.42±11.21 | 79.69±11.12 | 79.57±11.02 |
| HR | 84.51±8.42[a] | 81.15±9.78[b] | 79.5±9.71[bc] | 77.83±9.8[c] | 78.81±9.4[bc] |
| RR | 31.98±11.02[a] | 25.22±10.32[b] | 22.27±10.22[c] | 19.02±8.65[d] | 18.54±8.73[d] |

$T_{db}$, indoor dry-bulb temperature; RH, indoor relative humidity; HR, heart rate; RR, respiration rate.

0h, feeding time; 0.5h, 0.5 hour after feeding;1h, 1 hour after feeding; 1.5h, 1.5 hour after feeding; 2h, 2 hour after feeding.

a–d, Means in a row with different superscripts differ significantly ($P < 0.05$)

**Figure. S1**

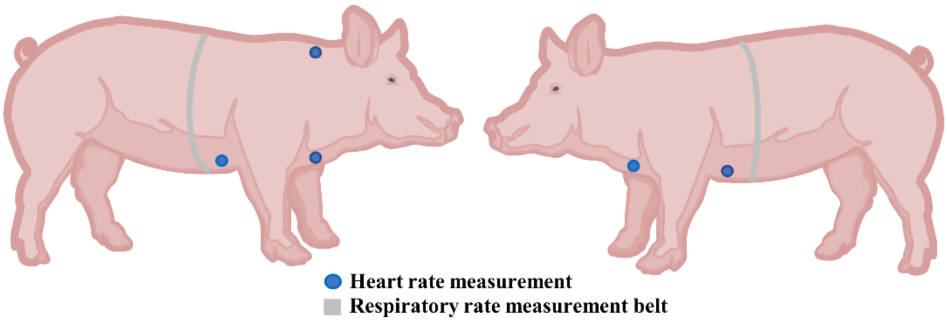


**Supplemental Figure S1.** Depiction of locations of heart rate (HR, bpm), and respiratory rate(RR, brpm) me asurements. The HR and RR was measured with JET device (Data Sciences International, St. Paul, MN, US A).
